# Ranking of Reactions Based on Sensitivity of Protein Noise Depends on the Choice of Noise Measure

**DOI:** 10.1371/journal.pone.0143867

**Published:** 2015-12-01

**Authors:** Sucheta Gokhale, Chetan Gadgil

**Affiliations:** 1 Chemical Engineering and Process Development Division, CSIR-National Chemical Laboratory, Pune, India; 2 CSIR-Institute of Genomics and Integrative Biology, Delhi, India; University of the Basque Country, SPAIN

## Abstract

Gene expression is a stochastic process. Identification of the step maximally affecting noise in the protein level is an important aspect of investigation of gene product distribution. There are numerous experimental and theoretical studies that seek to identify this important step. However, these studies have used two different measures of noise, viz. coefficient of variation and Fano factor, and have compared different processes leading to contradictory observations regarding the important step. In this study, we performed systematic global and local sensitivity analysis on two models of gene expression to investigate relative contribution of reaction rate parameters to steady state noise in the protein level using both the measures of noise. We analytically and computationally showed that the ranking of parameters based on the sensitivity of the noise to variation in a given parameter is a strong function of the choice of the noise measure. If the Fano factor is used as the noise measure, translation is the important step whereas for coefficient of variation, transcription is the important step. We derived an analytical expression for local sensitivity and used it to explain the distinct contributions of each reaction parameter to the two measures of noise. We extended the analysis to a generic linear catalysis reaction system and observed that the reaction network topology was an important factor influencing the local sensitivity of the two measures of noise. Our study suggested that, for the analysis of contributions of reactions to the noise, consideration of both the measures of noise is important.

## Introduction

Gene expression is known to be a stochastic process [1] and various aspects of the stochasticity in gene expression such as its origin, mechanisms for controlling stochasticity and effects of stochasticity on the outcome of gene expression have been studied since many years. One aspect is identification of the step in gene expression that maximally affects the ‘noise’ in the protein level. There are a number of experimental as well as theoretical studies seeking to identify the most important step. The major outcome of the studies is summarized in Table 1. However, the conclusions reached by these studies are contradictory. In order to analyse the effect of transcription and translation on noise in protein level, transcription and translation efficiencies were compared both theoretically [2] and experimentally[3]. Both the studies suggested that the noise in protein level was maximally influenced by translation. On the other hand, Blake, Kærn et al [4] compared transcription focusing on initiation, and translation in yeast cells. The study stated that as opposed to prokaryotes, transcription with re-initiation affects the noise in protein level in case of eukaryotic cells. Another experimental study investigated mRNA level variability in yeast population [5]. The study compared gene activation, deactivation, and transcription under three different conditions of specific rates of these reactions. The study suggested that infrequent gene activation and high transcription produced large variability in the population. A theoretical study by Komorowski, Mikisz et al [6] investigated the contributions of protein and RNA regulatory factors and stated that repression at translational level results in more noise than repression at transcription. In all these studies, the Fano factor (FF),defined as the ratio of protein number variance to mean, is used as the sole noise measure. The study by Raj, Peskin et al [7] to estimate the contributions of intrinsic and extrinsic noise sources to variability in mRNA population in mammalian cells stated that infrequent gene activation-deactivation determines the variability. A theoretical study by Kierzek, Zaim et al[8] in prokaryotic system compared transcription and translation, using coefficient of variation (CV),defined as the ratio of standard deviation to mean, as the measure of noise. The study concluded that transcription initiation frequency affects noise in the steady state protein level more than translation initiation frequency. It is evident that these studies have compared different processes and have also used two different measures of noise viz., CV and FF. A recent study analysing DNA looping has shown that the presence of looping always increases the CV but may decrease or increase the FF[9].This observation indicates the importance of considering both the measures of noise. The CV represents an inverse signal to noise ratio. On the other hand, FF describes the fluctuations relative to that of a Poisson distribution. Due to the use of different measures of noise in these studies, a comparison to draw a consistent conclusion about the step in gene expression maximally affecting the noise at steady state protein level is not feasible. This suggests a need for development ofa methodology of estimating the contribution of individual processes to the ‘noise’ in the expressed protein concentration and its implementation using the two different noise measures to identify the step in the protein expression process that maximally affects the noise (as defined by each measure) in steady state protein concentration. Here, we introduce the use of a global and local sensitivity analysis to estimate the relative sensitivity of model parameters to noise as defined by these two measures.

**Table 1:** Studies analysing gene expression show important step determining noise in protein.

Reference	Organism	Measure of noise	Study conclusion
Thattai and Van Oudenaarden 2001; Ozbudak, Thattai et al. 2002 [2, 3]	Prokaryote	Fano Factor	Translation
Blake, Kærn et al. 2003 [4]	Eukaryote	Fano factor	Transcription with re-initiation
Kierzek, Zaim et al. 2001 [8]	Prokaryote	Coefficient of variation	Transcription
Komorowski, Mikisz et al. 2009 [6]	Not specified (Theoretical)	Fano Factor	Translation

Theoretically, the dependence of any outcome on a particular reaction can be estimated by calculating its sensitivity to the reaction rate constants that define the rate of the reaction. Such sensitivity can be local or global. Local sensitivity quantifies the effect of an infinitesimal change in the reaction rate constant from a specified value, for a particular point in concentration state space, i.e. in a local region near a particular value of the concentration of all species in the reaction network. Local sensitivity of steady state outputcan be estimated by analytically or numerically calculating scaled or un-scaled partial derivative of the output with respect to a reaction rate constant. However, in order to reach a more general conclusion regarding the effect of the reaction rate a measure of the global sensitivity of output to changes in the parameter seems more appropriate. Such a global sensitivity captures the overall sensitivity to the parameter.It does not depend on any particular point in the concentration state space, and does not require the parameter change to be infinitesimal.

Sensitivity analysis has been performed for discrete stochastic systems using various approaches. One of the initial studies [10] obtained expression for local sensitivity of concentration correlation function for the systems represented by stochastic differential equations with additive or multiplicative white noise. Later studies reported sensitivity analysis for stochastic systems based on sensitivity of probability density function[11] or other approaches such as spectral polynomial chaos expansion[12], stochastic control analysis (SCA) framework[13], summation theorem[14], common reaction path method [15], and pathwise derivative approach [16]. All these methods identify local sensitivity for infinitesimally small or finite perturbations around a nominal parameter value.On the other hand, a study by Degasperi and Gilmore [17] used histogram difference method for global sensitivity analysis.A recent study by DhananjaneyuluV., G. Kumar, et al performed global sensitivity analysis for assessing the sensitivity of noise in a MAPK cascade[18].

It is known that in case of gene expression, the parameter values for each of the major steps span 2 to 3 orders of magnitude [19]. To examine sensitivity of these steps on the steady state noise in protein level global sensitivity analysis is the appropriate approach. As a first step to perform sensitivity analysis different sampling techniques such as full factorial sampling, Latin hypercube sampling, and random sampling are available. Various global sensitivity analysis methods such as multiple parameter sensitivity analysis (MPSA), partial rank correlation coefficient, Morris method, weighted average of local sensitivities,and variance based methods such as SOBOL, FAST, RS-HDMR are available [20].

In this study we performed a global sensitivity analysis to identify the relative contribution of each step in gene expression to steady state noise in protein level. We calculated global sensitivities of reactions to noise as quantified by both the measures of noise, viz., CV and FF. We used Latin hypercube sampling and estimated the global sensitivity using MPSA method. To identify whether different abstractions of gene expression lead to difference in sensitivities, we used two models of gene expression having different degree of details. An interesting fact evident from the analysis was that the two measures of noise were sensitive to different reaction parameters. CV was observed to be most sensitive to transcription and protein degradation in the two models of gene expression while FF was observed to be most sensitive to translation in both the models. We also obtained analytical expression for local sensitivity of these measures of noise. It explained the observed distinct sensitivities of the two measures of noise for the same parameters. We extended this analysis to a generic linear catalysis reaction system. From global sensitivity analysis of this system, we observed that in contrast to gene expression, CV was affected by all the reactions to similar extent. FF was observed to be sensitive to both catalysis and degradation reactions. From numerical calculation of local sensitivity coefficients at random parameter values, we observed that CV was maximally sensitive to the first catalysis reaction while FF was maximally sensitive to the last catalysis reaction. Thus, the reaction network topology was identifiedto be an important factor influencing the ranking of reactions based on their effect on local sensitivity of the two measures of noise.

## Methodology

### Gene expression models and parameter ranges

**Fig 1:**
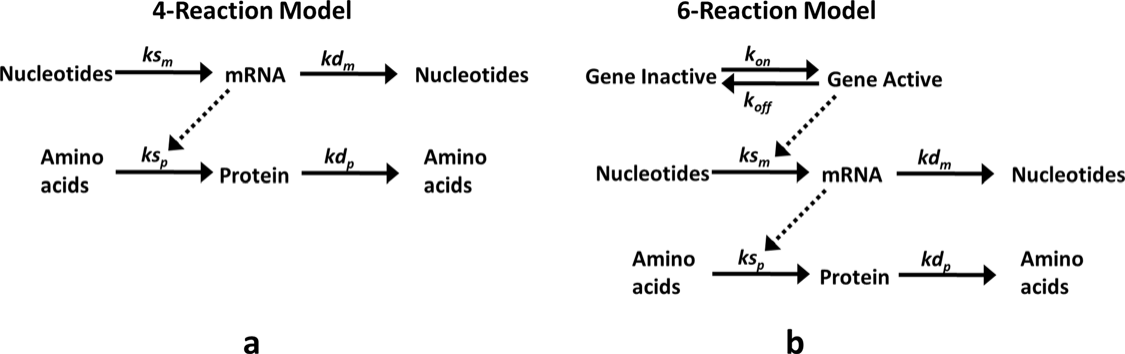
Figure showing reaction representation of 4-reaction (a) and 6-reaction (b) model of gene expression.

Global sensitivity analysis is performed using two models of gene expression viz., 4-reaction model (Fig 1A) and 6-reaction model (Fig 1B), having different levels of details.

The 4-reaction model isa widely used model of gene expression. It contains zero order synthesis of mRNA, first order synthesis of protein with rate proportional to mRNA level, and first order degradation of mRNA and protein. In the 6-reaction model, two additional reactions of gene activation and deactivation are included. In this model, mRNA synthesis isrepresented as a first order reaction with rate proportional to level of active gene. As a representative parameter range of eukaryotic gene expression, the parameter ranges for specific rates of transcription, translation, mRNA degradation and protein degradation are obtained from a data set of genome wide measurement bySchwanhausser, Busse et al [19]. In this dataset, a majority of the parameter values are observed to be centered on a mean value and a very few values lie at the boundaries. Consideration of the entire range would result in altered sensitivity due to outlier parameter values. Therefore, instead of considering the complete range for each parameter, log transformed mean ± 2 standard deviation range is considered. More than 95% of the data points are covered in the selected range. The ranges for specific rate of gene activation and deactivation are obtained from experimental study by Suter, Molina et al[21]. The parameter ranges are listed in Table 2.

**Table 2:** The parameter ranges used in gene expression models.

Parameter	Range of parameter values
Gene activation (min^-1^)	1*10^−1^–2*10^−3^
Gene inactivation (min^-1^)	1–2*10^−3^
Transcription reaction rate constant (min^-1^, for 4-reaction model)	4.33*10^−3^–2.16*10^−1^
mRNA degradation reaction rate constant (min^-1^)	2.98*10^−3^–4.80*10^−4^
Translation reaction rate constant (min^-1^)	8.10*10^−2^–37.67
Protein degradation reaction rate constant (min^-1^)	1.83*10^−3^–3.82*10^−5^

In case of 6-reaction model, the specific rate of mRNA synthesis is calculated using the specific rates of gene activation, deactivation and specific rate of mRNA synthesis for 4-reaction model. Knowing the values of gene activation and deactivation reaction rate parameters the steady state active gene level is calculated and used to set the specific rate of mRNA synthesis in order to obtain the same steady state rate of mRNA synthesis as that would be obtained in case of 4-reaction model.

$$ks_{m\_6-reaction}\times {\left[g_{on}\right]}_{steady\_state}=ks_{m\_4-reaction}$$

Therefore, the reaction rate parameter for mRNA synthesis in case of 6-reaction model isgivenas Eq 1,

$$ks_{m\_}{}_{6-reaction}=\frac{ks_{m\_}{}_{4-reaction}}{\frac{k_{on}}{\left(k_{on}+k_{off}\right)}}$$ (1)

### Calculation of steady state Coefficient of Variation and Fano factor

Using the frameworkfor the expression for the time evolution of the moments derived using exact stochastic chemical master equation described previously[22]for first order reactions, the differential equations for the time evolution of first and second moments are obtained for 4-reaction and 6-reaction models as follows.Considering the 4-reaction gene expression model as one step catalytic reaction system following matrices are obtained.

$$K_{4}{}^{s}=\left[\begin{array}{cc} ks_{m} & 0\\ 0 & 0 \end{array}\right],K_{4}{}^{d}=\left[\begin{array}{cc} kd_{m} & 0\\ 0 & kd_{p} \end{array}\right],K_{4}{}^{cat}=\left[\begin{array}{cc} 0 & 0\\ ks_{p} & 0 \end{array}\right],k_{4}{}^{con}=0$$

$$K_{4}=K_{4}{}^{cat}+K_{4}{}^{con}-K_{4}{}^{d}=\left[\begin{array}{cc} -kd_{m} & 0\\ ks_{p} & -kd_{p} \end{array}\right]$$

The time evolution of mean (M_4_) is given by Eq 2,

$$\frac{dM_{4}(t)}{dt}=\frac{d}{dt}\left[\begin{array}{c} m(t)\\ p(t) \end{array}\right]=K_{4}M_{4}(t)+K_{4}{}^{s}1$$ (2)

The time evolution of second moment is given by Eq 3,

$$\frac{dV_{4}(t)}{dt}=K_{4}V_{4}(t)+{\left(K_{4}V_{4}(t)\right)}^{T}+\Gamma_{4}+\Gamma_{4}{}^{T},\;\text{where}\;\Gamma_{4}{}_{ij}=\left(K_{4}{{}^{cat}}_{ij}\_K_{4}{{}^{s}}_{ii}\right)M_{4}{}_{j}$$ (3)

Solving the differential equations for moments at steady state, expressions for the steady state mean and variance for mRNA and protein are obtained.

For 6-reaction system, using the same framework, following matrices are obtained.

$$K_{6}{}^{s}=0,K_{6}{}^{d}=\left[\begin{array}{cccc} 0 & 0 & 0 & 0\\ 0 & 0 & 0 & 0\\ 0 & 0 & kd_{m} & 0\\ 0 & 0 & 0 & kd_{p} \end{array}\right],K_{6}{}^{cat}=\left[\begin{array}{cccc} 0 & 0 & 0 & 0\\ 0 & 0 & 0 & 0\\ 0 & ks_{m} & 0 & 0\\ 0 & 0 & ks_{p} & 0 \end{array}\right],K_{6}{}^{con}=\left[\begin{array}{cccc} -k_{on} & k_{off} & 0 & 0\\ k_{on} & -k_{off} & 0 & 0\\ 0 & 0 & 0 & 0\\ 0 & 0 & 0 & 0 \end{array}\right]$$

$$K_{6}=K_{6}{}^{cat}+K_{6}{}^{con}-K_{6}{}^{d}=\left[\begin{array}{cccc} -k_{on} & k_{off} & 0 & 0\\ k_{on} & -k_{off} & 0 & 0\\ 0 & ks_{m} & -kd_{m} & 0\\ 0 & 0 & ks_{p} & -kd_{p} \end{array}\right]$$

The time evolution of mean (M_6_) is given by Eq 4,

$$\frac{dM_{6}(t)}{dt}=\frac{d}{dt}\left[\begin{array}{c} g_{off}\\ g_{on}\\ m\\ p \end{array}\right]=K_{6}M_{6}(t)$$ (4)

The time evolution of second moment is as given in Eq 5,

$$\frac{dV_{6}(t)}{dt}=K_{6}V_{6}(t)+{\left(K_{6}V_{6}(t)\right)}^{T}+\Gamma_{6}+\Gamma_{6}{}^{T}$$ (5)

where $\Gamma_{6}{}_{ij}=\left(K_{6}{{}^{cat}}_{ij}\right)M_{6}{}_{j}$

The equations for both the models are numerically integrated using Matlab differential equation solver ode15s. Using the steady state values of the two moments, steady state CV and FF for mRNA and protein level are calculated.Stochastic simulations of the gene expression models are performed using exact stochastic simulation algorithm [23], implemented in Fortran. The output of numerical simulations is in agreement with the output of moment differential equations (results not shown).

### Global sensitivity analysis

Global sensitivity analysis of CV and FFis performed using Multiple Parameter Sensitivity Analysis (MPSA). A sample of 100 parameter sets is generated using Latin hypercube samplingto calculate sensitivity.Matlab function lhsdesign is used to generate the samples.We verifiedthat change in the sample size did not result in qualitative change in sensitivity output. In case of 4-reaction model, a parameter set contained 4 parameters(specific rates of mRNA and protein synthesis and degradation) in the range stated in Table 2. In case of 6-reaction model, a parameter set contained 6 parameters, the four above and2 additional parameters for gene activation and deactivation.%CV (CV*100) and FFvalues for each of the parameter sets are calculated. Depending upon the CV or FF values of the parameter sets being lower or higher than that of the reference parameter set, the parameter sets are classified into two classes.As the parameter sets are randomly generated first parameter set is considered as a reference parameter set. We verifiedthat selecting any parameter set as a reference resulted into qualitatively same results. Parameter sensitivity is determined by comparing the cumulative distribution function curves for each parameter sets using Kolmogorov-Smirnov test. The test is performed using Matlab function kstest2. To measure the sensitivity of each parameter a score is defined by considering the test statistics weighted by the negative logarithm of p-value. The score is defined ask*(-log_*10*_ *(p))*, where *k* is KS test statistics and *p* is p-value of the test. The sensitivity score is calculated for asample. The average score of the 5 samples is used as a measure of sensitivity. We verified that reducing the number of samples does not change the ranking of reactions.

### Analytical expression for local sensitivity

To understand the contribution of reaction rate parameters to sensitivity of steady state CV and FF, analytical expressions for local sensitivity are obtained. Local sensitivity is defined as the change in the model output relative to infinitesimally small change in parameter value i.e. *δ*(*output*)/*δp*_*i*_. The 4-reaction model of gene expression is considered as a one-step linear catalysis model, and analytical expression for steady state protein CV and FFis obtained as mentioned. Sensitivity coefficients are calculated by obtaining the partial derivatives of CV and FF with respect to every parameter. Relative sensitivity coefficients $\frac{\delta (output)}{\delta p}.\frac{p}{output}$ are calculated to compare the sensitivity values at different parameter values. In the 4-reaction model, parameters *ks*_*m*_, *kd*_*m*_, *ks*_*p*_, and *kd*_*p*_are defined as specific rates of mRNA synthesis, mRNA degradation, protein synthesis, and protein degradation, respectively. The expressions for sensitivity coefficients, and normalized sensitivity coefficient are obtained using Mathematica version 7.0.1.0 (Wolfram Research, Champaign, USA).

### Sensitivity analysis for generic linear catalysis cascades

**Fig 2:**
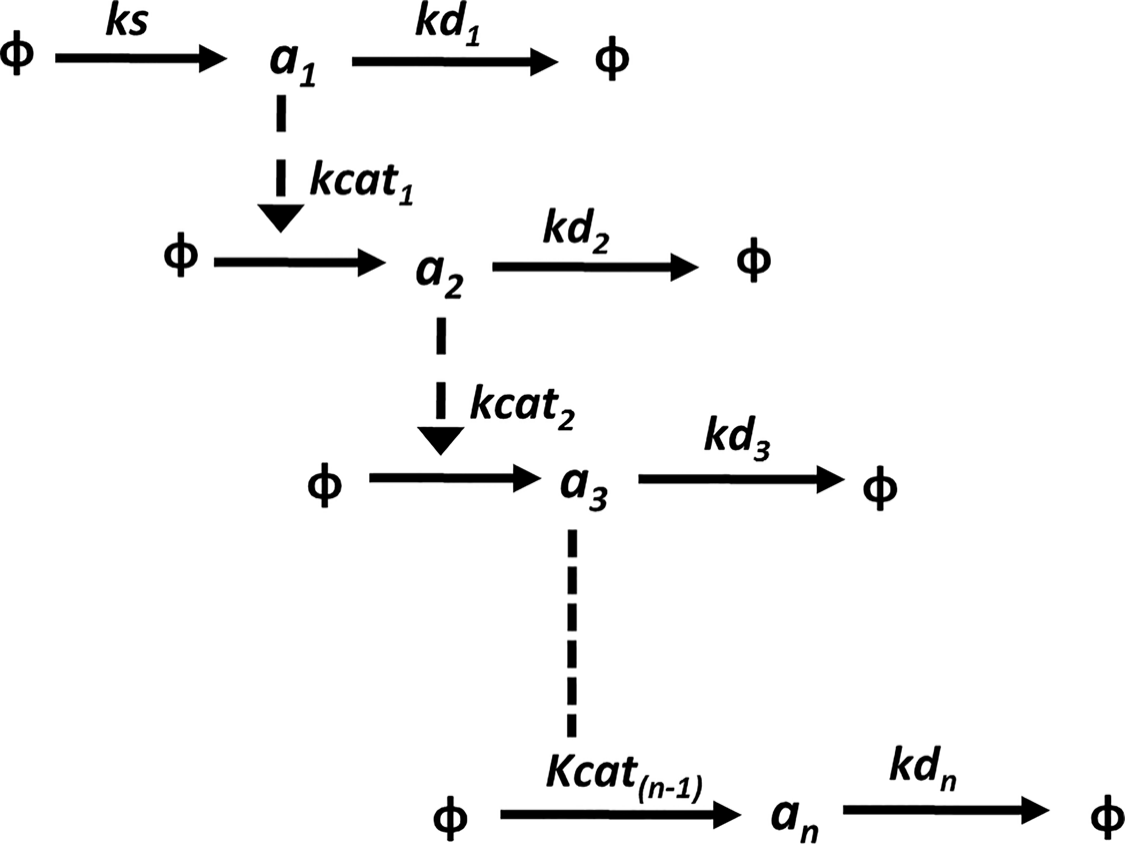
A generic linear catalysis reaction system. The parameter for the first zero order synthesis reaction is referred as *ks*, the catalysis reaction rate parameters are referred as *kcat*_*1*_ to *kcat*_*(n-1)*_, and degradation reaction rate parameters are referred as *kd*_*1*_ to *kd*_*n*_

The reaction system of a generic cascade of linear catalysis reactions is represented in Fig 2.

Using the differential equations for the time evolution of the moments, the steady state CV and FF for the last (n^th^) component in a generic (n-step) linear catalysis cascade is calculated numerically. Similar to the gene expression models, global sensitivity analysis is performed using MPSA for cascades of length up to 5 steps. In this case an arbitrary range of 10^−2^ to 10^2^is considered for specific rates of all the three reaction types viz., synthesis, catalysis, and degradation. 5 samples of 100 parameter sets each are generated to calculate sensitivity. The average score is considered as a measure of sensitivity.

For local sensitivity analysis same parameter range of 10^−2^ to 10^2^is considered. Local sensitivity coefficient for each reaction rate parameter is calculated numerically by perturbing the parameter value randomly generated within the range by 1% of its nominal value. The frequency of every reaction parameter of having highest value of local sensitivity coefficient as compared to other reaction rate parameters is calculated. Assuming equal sensitivity to all reactions, the obtained frequency is compared with the expected equal frequency using Chi-square test of independence.

## Results

### Steps in gene expression affect different measures of noise to different extent

**Fig 3:**
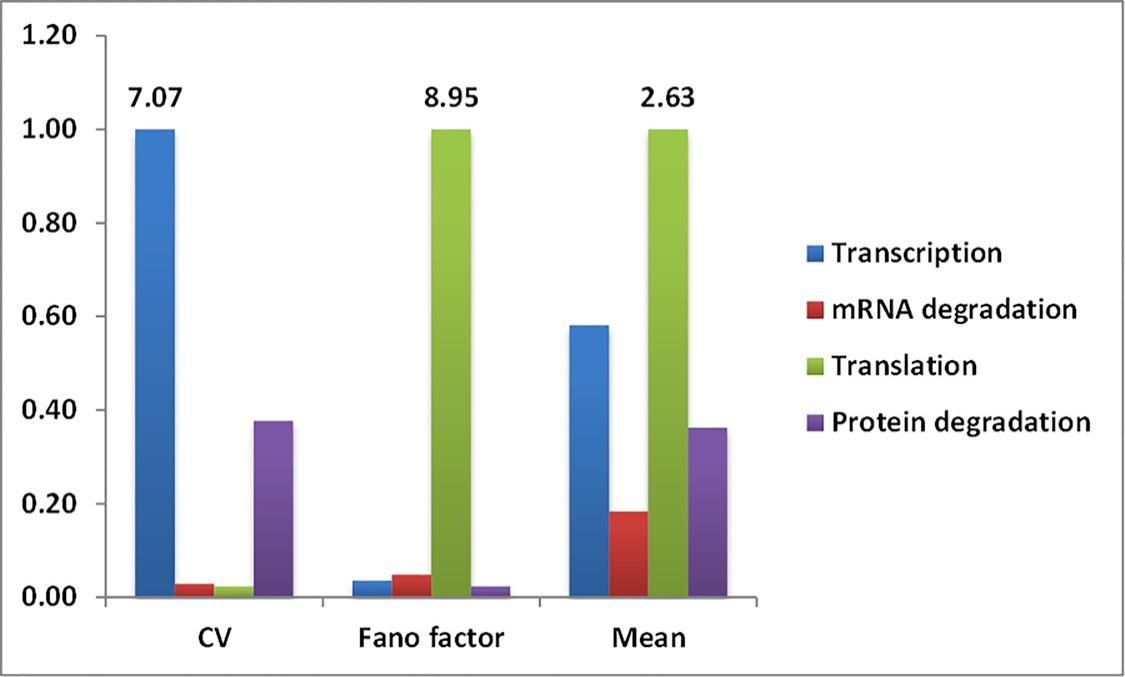
Bar plot of normalized sensitivity scores for CV, FF, and meanof steady state protein level for reaction rate parameters of 4-reaction model of gene expression. Data labels on the top of bars are the sensitivity scores.

To identify the step in gene expression that maximally influences noise at steady state protein level, global sensitivity analysis is performed using MPSA method, as described in the methods section. The scaled sensitivity score is used as a measure of the relative sensitivity of each reaction parameter. Initially, a widely used 4-reaction model of gene expression is used. It includes zero order synthesis of mRNA, first order synthesis of protein with rate dependent on mRNA level, and first order degradation reactions for mRNA and protein. The ranking of reactions based on normalized sensitivity score is shown in Fig 3. The average sensitivity scores for this model are given in Table A in S1 File. Interestingly, from the sensitivity scores it is observed that the two measures of noise show distinct sensitivities for same reaction parameters. CVis observed to be most sensitive to transcription while FFis observed to be most sensitive to translation.The ranking of reaction parameters according to sensitivity did not changeeven with a different sample size

It is evident thatCVis sensitive to protein degradation to some extent but much less sensitive to mRNA degradation and even translation. FF, on the other hand, is observed to be much less sensitive to all the other three steps compared to translation. Analytical expressions for local sensitivity of these measures of noise with respect to the four parameters explained the distinct contribution of parameters.

**Fig 4:**
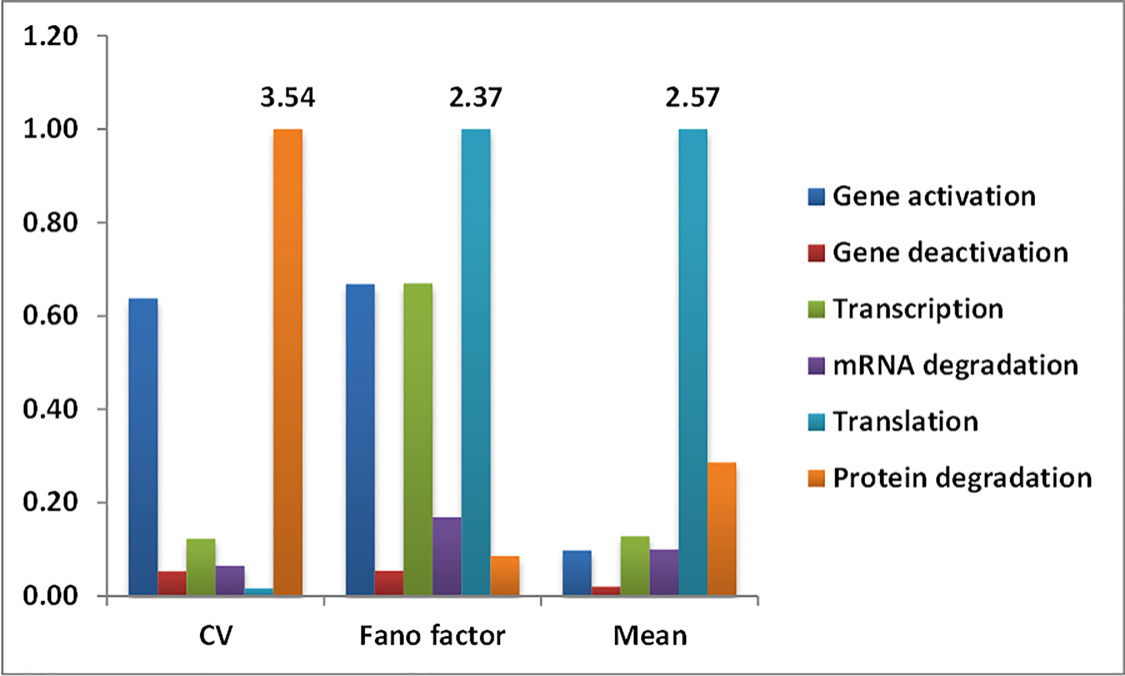
Bar plot of normalized sensitivity scores for CV, FF, and mean of steady state protein level for reaction rate parameters of 6-reaction model of gene expression. Data labels on the top of bars are the sensitivity scores.

The experimental studies have compared different processes at a time such as transcription and translation or gene activation, deactivation and transcription. Such pair-wise comparisonof different processes due to experimental constraints makes it difficult to obtain a global understanding of the relative sensitivity of noise to each individual process. Therefore, we performed similar global sensitivity analysis on a model which contains gene activation and deactivation reactions in addition to the 4 reactions of mRNA and protein synthesis and degradation. Comparison of obtained sensitivity can indicate whether considering different levels of details change the conclusions about the relative ranking of processes based on their role in influencing expression noise. Additionally, the 6-reaction model contains all the major steps that are considered in the previous studies. The ranking of reactions based on sensitivity scores is represented in Fig 4. The absolute values of sensitivity scores are given in Table C in S1 File. Similar to the 4-reaction model, in this casetoo the two measures of noise show distinct sensitivities to the reaction rate parameters. It is observed that, addition of reactions changed the relative contribution of steps to the steady state noise.

In contrast to four reaction model where CVis observed to be most sensitive to transcription, in this case protein degradation is observed to maximally affect CV. It is also observed to be sensitive to gene activation though to a slightly lesser extent. However, gene deactivation, transcription, mRNA degradation and translation are observed to affect CV to much lesser extent. The relative ranking of processes depending on their contribution to sensitivity of FF did not change with increase in model complexity. Similar to the four reaction model, FFis observed to be most sensitive to translation for 6-reaction model as well. In contrast to four reaction model, where other reaction parameters are observed to affect FF to much lesser extent, in this case gene activation, and transcription are also observed to affect FF.

In summary, it is observed that reaction rate parameters affect the two measures of noise to different extents, and this ranking also depends on the mathematical model for the gene expression process when CV is used as the noise measure. CV for steady state protein level is observed to be sensitive to transcription in 4-reaction model of gene expression, while it is observed to be most sensitive to protein degradation in case of 6-reaction model. FFis always observed to be most sensitive to translation.To our knowledge, except for signalling systems [24] previous gene expression studies have not analysed the effect of variation of mRNA and protein half- life on the steady state variability of protein.In this study protein half-lifeis observed to be important to affect the CV at steady state protein level.

In addition to the sensitivity for two measures of noise, sensitivity score is calculated for mean level. Protein steady state mean level is observed to be most sensitive to translation for both 4 and 6-reaction models, similar to that observed for FF. Previous studies have suggested orthogonal (independent) control of mean and noise in biological systems [2, 25]. In this case, change in translation to change mean level is observed to affectFF as well. But CVis changed to a very lesser extent. While change in transcription or protein degradation is observed to change CV but affects mean protein level and FF to a very lesser extent. Therefore, the orthogonal control of mean and noise is observed to be possible only under certain conditions.

### Analytical expression for local sensitivity identifiesdistinct contributions of parameters to the two measures of noise

To examine the contribution of the reaction rate parameters to sensitivity of these two measures of noise, analytical expressions for local sensitivity are obtained. Although local sensitivity is useful to investigate the sensitivity of the output to a parameter around its nominal value, the analytical expression would be informative in order to examine the sensitivity as a function of the parameters.

To understand the contributions of each reaction rate parameter to CV and FF, analytical expression for these two measures of noise is obtained for 4-reaction model of gene expression. The expressions (details in S1 File) for CVand FF are given by Eqs 6 and 7 respectively.

$$CV=\sqrt{\frac{kd_{m}kd_{p}(kd_{m}+ks_{p}+kd_{p})}{ks_{m}ks_{p}(kd_{m}+kd_{p})}}$$ (6)

$$Fano\;factor=\frac{kd_{m}+ks_{p}+kd_{p}}{kd_{m}+kd_{p}}$$ (7)

Here, *ks*_*m*_, *kd*_*m*_, *ks*_*p*_, and *kd*_*p*_ represent specific rates of mRNA synthesis, mRNA degradation, protein synthesis, and protein degradation respectively. From the analytical expressions (Eqs 6 and 7) it is observed that all the four parameters contribute to the steady state CV while, transcription does not contribute to FF at steady state protein level. Therefore, change in specific rate of transcription would not affect FF at steady state protein level. From the numerical global sensitivity analysis transcription is observed to have very less sensitivity score for transcription. The observed sensitivity value can be due to simultaneous variation in parameters performed for global sensitivity.

The analytical expressions for sensitivity of CV with respect to each of the four parameters are obtained as the partial derivatives of Eq 6 as Eqs 8–11.

$$\mathrm{Sensitivity of CV to transcription}=\frac{\delta CV}{\delta ks_{m}}=-\frac{kd_{m}kd_{p}(kd_{m}+ks_{p}+kd_{p})}{2ks_{m}{}^{2}ks_{p}(kd_{m}+kd_{p})\sqrt{\frac{kd_{m}kd_{p}(kd_{m}+ks_{p}+kd_{p})}{ks_{m}ks_{p}(kd_{m}+kd_{p})}}}$$ (8)

$$\mathrm{Sensitivity of CV to mRNA degradation}=\frac{\delta CV}{\delta kd_{m}}=\frac{\frac{kd_{m}kd_{p}}{ks_{m}ks_{p}(kd_{m}+kd_{p})}-\frac{kd_{m}kd_{p}(kd_{m}+ks_{p}+kd_{p})}{ks_{m}ks_{p}{\left(kd_{m}+kd_{p}\right)}^{2}}+\frac{kd_{p}(kd_{m}+ks_{p}+kd_{p})}{ks_{m}ks_{p}(kd_{m}+kd_{p})}}{2\sqrt{\frac{kd_{m}kd_{p}(kd_{m}+ks_{p}+kd_{p})}{ks_{m}ks_{p}(kd_{m}+kd_{p})}}}$$ (9)

$$\mathrm{Sensitivity of CV to translation}=\frac{\delta CV}{\delta ks_{p}}=\frac{\frac{kd_{m}kd_{p}}{ks_{m}ks_{p}(kd_{m}+kd_{p})}-\frac{kd_{m}kd_{p}(kd_{m}+ks_{p}+kd_{m})}{ks_{m}ks_{p}{}^{2}(kd_{m}+kd_{p})}}{2\sqrt{\frac{kd_{m}kd_{p}(kd_{m}+ks_{p}+kd_{p})}{ks_{m}ks_{p}(kd_{m}+kd_{p})}}}$$ (10)

$$\mathrm{Sensitivity of CV to protein degradation}=\frac{\delta CV}{\delta kd_{p}}=\frac{\frac{kd_{m}kd_{p}}{ks_{m}ks_{p}(kd_{m}+kd_{p})}-\frac{kd_{m}kd_{p}(kd_{m}+ks_{p}+kd_{p})}{ks_{m}ks_{p}{\left(kd_{m}+kd_{p}\right)}^{2}}+\frac{kd_{m}(kd_{m}+ks_{p}+kd_{p})}{ks_{m}ks_{p}(kd_{m}+kd_{p})}}{2\sqrt{\frac{kd_{m}kd_{p}(kd_{m}+ks_{p}+kd_{p})}{ks_{m}ks_{p}(kd_{m}+kd_{p})}}}$$ (11)

From these expressions it is observed that, CV decreases by increasing specific rate of mRNA synthesis or protein synthesis. However, such relationship is not observed for specific rates of mRNA and protein degradation. It is evident by comparing the expressions for sensitivity of CV for mRNA degradation, translation and protein degradation (Eqs 9, 10 and 11) that the expressions for sensitivity to mRNA degradation and protein degradation contain additional positive terms $\frac{kd_{p}(kd_{m}+ks_{p}+kd_{p})}{ks_{m}ks_{p}(kd_{m}+kd_{p})}$ and $\frac{kd_{m}(kd_{m}+ks_{p}+kd_{p})}{ks_{m}ks_{p}(kd_{m}+kd_{p})}$ in the numerator respectively. Therefore, the sensitivity of CV to mRNA degradation and protein degradation is greater than that for translation at any given value of parameters. Generally mRNA molecules are known to be less stable than protein molecules, indicating higher numerical values of mRNA degradation reaction rate constant than protein degradation. Therefore, comparing the expression for sensitivity to mRNA degradation and protein degradation, it can be inferred that sensitivity of CV to mRNA degradation would be lesser than that for protein degradation for majority of the cases, as observed in numerical sensitivity analysis. However, only in case of a very stable mRNA and unstable protein it may not hold true. Overall, for CV as a noise measure, no conclusion about the relative sensitivity ranking can be drawn that is independent of theparameter values.

The analytical expressions for sensitivity of FF to the four parameters are similarly obtained as the partial derivatives of Eq 7. The analytical expressions are given as Eqs 12–15.

$$\mathrm{Sensitivity of Fano factor to transcription}=\frac{\delta \mathrm{Fano factor}}{\delta ks_{m}}=0$$ (12)

$$\mathrm{Sensitivity of Fano factor to mRNA degradation}=\frac{\delta \mathrm{Fano factor}}{\delta kd_{m}}=\frac{1}{kd_{m}+kd_{p}}-\frac{kd_{m}+ks_{p}+kd_{p}}{{\left(kd_{m}+kd_{p}\right)}^{2}}$$ (13)

$$\mathrm{Sensitivity of Fano factor to translation}=\frac{\delta \mathrm{Fano factor}}{\delta ks_{p}}=\frac{1}{kd_{m}+kd_{p}}$$ (14)

$$\mathrm{Sensitivity of Fano factor to protein degradation}=\frac{\delta \mathrm{Fano factor}}{\delta kd_{p}}=\frac{1}{kd_{m}+kd_{p}}-\frac{kd_{m}+ks_{p}+kd_{p}}{{\left(kd_{m}+kd_{p}\right)}^{2}}$$ (15)

From Eq 12, it is clear that specific transcription rate will always be the parameter for which the FF is least sensitive. From Eqs 13 and 15, it is clear that the sensitivity of FF to mRNA and protein degradation is the same, independent of the actual values of the four parameters. Comparing the two equations with Eq 14, itisobserved that the negative term $\frac{kd_{m}+ks_{p}+kd_{p}}{{\left(kd_{m}+kd_{p}\right)}^{2}}$ does not appear in Eq 14. Therefore, the sensitivity of FF to translation is greater than that for mRNA and protein degradation, independent of parameter values.Interestingly, it is observed that, sensitivity of FFto translation would depend only upon the value of mRNA and protein degradation and not on value of specific rate of translation.

As local sensitivity examines the behaviour of model in the neighbourhood of a nominal parameter values, the numerical value of sensitivity differs depending upon the actual parameter values. To compare local sensitivity at different parameter values, relative sensitivity coefficient is calculated.

The relative sensitivity coefficients for CV at steady state are given by Eqs 16–19.

$$\frac{\delta {CV}_{\mathrm{Protein}}}{\delta ks_{m}}.\frac{ks_{m}}{{CV}_{\mathrm{Protein}}}=-\frac{1}{2}$$ (16)

$$\frac{\delta CV_{\mathrm{Protein}}}{\delta kd_{m}}.\frac{kd_{m}}{CV_{\mathrm{Protein}}}=\frac{kd_{m}{}^{2}+2kd_{m}kd_{p}+kd_{p}{}^{2}+ks_{p}kd_{p}}{2(kd_{m}+kd_{p})(kd_{m}+ks_{p}+kd_{p})}$$ (17)

$$\frac{\delta CV_{\mathrm{Protein}}}{\delta ks_{p}}.\frac{ks_{p}}{CV_{\mathrm{Protein}}}=\frac{-(kd_{m}+kd_{p})}{2(kd_{m}+ks_{p}+kd_{p})}$$ (18)

$$\frac{\delta CV_{\mathrm{Protein}}}{\delta kd_{p}}.\frac{kd_{p}}{{CV}_{\mathrm{Protein}}}=\frac{kd_{m}{}^{2}+2kd_{m}kd_{p}+kd_{p}{}^{2}+ks_{p}kd_{m}}{2(kd_{m}+kd_{p})(kd_{m}+ks_{p}+kd_{p})}$$ (19)

From Eq 16, it is evident that the relative change in CV for change in transcription at any parameter values is same and other parameters do not show any effect. From Eq 18, it can be inferred that at very low translation compared to mRNA and protein degradation, for ∆ increase in parameter, there would be 0.5∆ decrease in scaled CV. While at high translation, mRNA and protein degradation would not affect and the change in CV would depend upon the specific rate of translation.

Similarly, to compare the sensitivity of FF, relative sensitivity coefficient is calculated as given by Eqs 20–22

$$\frac{\delta FF_{\mathrm{Protein}}}{\delta kd_{m}}.\frac{kd_{m}}{FF_{\mathrm{Protein}}}=\frac{-kd_{m}ks_{p}}{\left(kd_{m}+kd_{p})(kd_{m}+ks_{p}+kd_{p}\right)}$$ (20)

$$\frac{\delta FF_{\mathrm{Protein}}}{\delta ks_{p}}.\frac{ks_{p}}{FF_{\mathrm{Protein}}}=\frac{ks_{p}}{kd_{m}+ks_{p}+kd_{p}}$$ (21)

$$\frac{\delta {FF}_{\mathrm{Protein}}}{\delta kd_{p}}.\frac{kd_{p}}{{FF}_{\mathrm{Protein}}}=\frac{-kd_{p}ks_{p}}{\left(kd_{m}+kd_{p})(kd_{m}+ks_{p}+kd_{p}\right)}$$ (22)

The expression for relative sensitivity coefficient for translation (Eq 21) indicates that at high values of specific rate of translation, the relative sensitivity coefficient would approach one.

### Distinct sensitivities of coefficient of variation and Fano factor are also evident in generic linear catalysis cascades

The observations about 4-reaction and 6-reaction model led to questions about sensitivity properties in generic linear catalysis cascades. We investigated whether the sensitivity properties observed in case of one step catalysis model i.e. 4-reaction model of gene expression were also observed for generic n-step catalysis. Using the differential equations for time evolution of moments for linear catalysis reactions [22] the steady state CV and FF for the n^th^ component are numerically calculated. MPSA is performed to calculate the sensitivity scores. Similar to the results for the 4- and 6-reaction gene expression models, global sensitivity analysis of a generic linear catalysis cascade shows distinct sensitivities of the two measures of noise to reaction rate parameters. However, in this case, much less difference is observed between the sensitivity values of CV for the reactions. For instance, the difference between lowest and highest sensitivity score is up to four fold, in contrast to more than one order of magnitude difference observed in case of gene expression models. Therefore, the observation of important steps such as gene activation, transcription or protein degradation is true only with the specific physiological parameter ranges. The FF, similar to gene expression models, is observed to be less sensitive to the first zero order synthesis reaction parameter. It is observed to be sensitive to both catalysis and degradation reactions. The sensitivity scores are given in S1 File. Local sensitivity analysis is also performed by numerically calculating sensitivity coefficients at random parameter values for generic linear catalysis cascades having length up to five steps. Assuming equal sensitivity to all the reactions, the observed frequency of each reaction parameter having highest sensitivity is compared with the expected equal frequency using Chi-square test of independence. In all the cases, the observed frequency of reactions having highest value of sensitivity coefficient is significantly different than the equal frequency for all reactions. In case of CV, for cascades of length greater than one, the frequency of first catalysis reaction having highest local sensitivity coefficient is observed to be maximum. In case of one-step cascade, last degradation reaction is observed to have maximum frequency of highest sensitivity coefficient for CV. In all the cascades, the frequency of last catalysis reaction having highest local sensitivity coefficient for FFis observed to be maximum. The details are given in S1 File.

## Discussion

In this study, we examined the sensitivity of two measures of noise to both local and global variations in the reaction rate parameters for models of gene expression and extended the analysis to a generic linear catalysis reaction system.

We varied the specific rates of reactions in gene expression model in wide ranges and performed sensitivity analysis to identify step(s) affecting the variability in protein level. Previous experimental studies have varied transcriptional and translational efficiencies or promoter state. In the experimental study by Ozbudak, Thattai et al [3] transcription rate was varied using two methods: varying the inducer concentration in the growth media in a system where transcription efficiency was controlled using an inducible promoter; and using single or double base pair mutations in the promoter region to control transcription efficiency. Translation efficiency was regulated by using mutant strains having mutations in ribosome binding site and initiation codon of reporter protein. In the context of a mathematical model for gene expression, these experimental changes are equivalent to a change in the transcription rate constant and translation rate constant respectively. In the study byBlake, Kærn et al [4] transcription was regulated by using two modes. In the native mode, naturally occurring GAL1 promoter was used which was regulated by modulating the concentration of galactose in the growth media. In the artificial mode, Tet-responsive GAL1 promoter was used and the TetR (Tet-repressor) mediated repression was relieved by modulating inducer ATC concentration. Translation efficiency was changed by generating codon variants having differing codon adaptation index. In the study by Raser, O'Shea [5], that considers contribution by gene activation and deactivation reaction, gene activation was regulated by changing promoter state using mutant strains that lack single component of chromatin remodelling complexes such as SNF/SWI, INO80, SAGA.In these studies, by regulating one reaction overall mRNA synthesis or protein synthesis is modulated. In the modeling study it can be equivalently represented by changing the specific reaction rate parameter of gene activation or mRNA or protein synthesis reaction. In this study, both the measures of noise, viz, CV and FFare used. The analysis revealed that steps in gene expression affect these two measures of noise to different extent. The steady state CVis observed to be most sensitive to transcription and protein degradation in 4-reaction and 6-reaction models, respectively,while the FFis observed to be most sensitive to translation in both the models of gene expression. Previous theoretical study on first order reaction network [22] and a recent study on DNA looping [9]have shown that the two measures lead to contrary conclusions about noise. The distinct contributions of reaction rate parameters are evident from the analytical expression for local sensitivity. The analytical expressions and the observed distinct sensitivity suggest, that the use of a particular measure affects the conclusions regarding the most important step in gene expression.

CV, defined as the ratio of standard deviation to mean, is a dimensionless quantity. It is used to compare variability in populations having different mean values. FF is the ratio of variance to mean. It can be indicative of nature of distribution, in particular distributions like Poisson for which variance and mean are equal.To identify the effect of variation of transcription or translation, use of CV would be useful as these reactions also affect the mean steady state level. FF should be used when it is appropriate to approximate the system to univariate discrete random process. For instance, in case of constitutively expressed or highly transcribed genes, gene expression can be represented as constant protein synthesis and first order protein degradation. In such conditions, the steady state protein distribution would be Poisson and therefore, FF would be important to consider. Therefore, appropriate context dependent use of a measure of noise is important. The sensitivity estimated using a particular measure of noise should not be attributed to the generic variability or ‘noise’. Considering the observations of this study, the data from the previous studies can be analysed using the other measure of noise as well, to examine whether the important step(s) in gene expression identified using one measure of noise remain the same when the other measure of noise is used. The results shown here suggest that for at least some cases, the relative importance of constituent steps to noise will change depending on the measure.

In this study, we obtained the analytical expressions for local sensitivity of CV and FF for steps in gene expression. Though local sensitivity analysis identifies sensitivity around one pointin the multi-dimensional parameter space, it helps to identify the contribution of each parameter to the sensitivity. Global sensitivity can be perceived as an average effect of local sensitivity function. In case of gene expression, global sensitivity indicates overall important step within the wide ranges of parameters, while local sensitivity would identify a step having maximum effect at given values of parameters. Therefore, the ranking of steps obtained by local sensitivity analysis can differ depending upon actual parameter values.

We extended the sensitivity analysis to generic cascades of linear catalysis reactions. It revealed that all the parameters affect the steady state CV of the last component to equal extend. The steady state FF of last component is observed to be least sensitive to the first zero order synthesis reaction. It is observed to be sensitive to both catalysis and degradation reactions to similar extent. Thus the observed high sensitivity of CV towards first synthesis reaction (mRNA synthesis) or last degradation reaction (protein degradation) in case of gene expression models can be due to the parameter ranges characteristic of synthesis and half-life of mRNA and protein. From local sensitivity analysis, the first catalysis reaction is observed to be important affecting CV maximally, while the last catalysis reaction is observed to affect FF maximally. Therefore, the topology is observed to be important to determine the local effect of parameters on CV and FF. Serial catalysis cascades are common in signalling systems such as MAPK cascades. Such systems can be analysedbased on the generic serial catalysis framework to explore the sensitivity properties.

Gene expression is a result of complex interactions between multiple molecular species. It has been shown that interactions with regulators, for instance, binding of transcription factor affects noise in its target gene expression [26–28]. Such systems with regulated synthesis reactions can also be analysed using the generic serial catalysis framework to examine the effects on the measures of noise.

The noise measures, CV and FF used in this study can be applied to reaction systems that lead to unimodaldistributions of component under investigation. For multimodal distribution use of these measures will lead to lose of information about the nature of distribution.The sensitivity analysis in this study, takes into account the sensitivity to the intrinsic noise. Extrinsicsources of noise such as variation in polymerase or ribosome numbers etc. are not considered. Additionally, the current framework is appropriate for the first order reactions. The current sensitivity analysis can be applied to pseudo-first order reaction systems where approximations of constant pool of transcription or translation machinery are appropriate.

Overall, thisstudy highlights that the analysis of important step in gene expression should consider various system specific factors such as the type of reaction system that is being analysed, and the nature of major sources of noise and suggests a context dependent use of measures of noise.

## Conclusion

In this study, we examined steps in gene expression that maximally affect the steady state protein level noiseby performing global sensitivity analysis. In contrast to the previous studies, we used both the measures of noise, viz., CV and FF. We observed that the steps in gene expression contribute differently to these two measures of noise, showing distinct relative sensitivities of these measures of noise to the same reaction rate parameter. The sensitivity analysis is performed using two models of gene expression having different levels of details. We observed that such addition of reactions changes the relative contribution towards the sensitivity. Analytical expressions for local sensitivity explained the observed distinct sensitivities for the same reaction parameter. From the sensitivity of mean level, it is observed that independent control of variability and mean level is possible only under certain conditions. We extended the sensitivity analysis to a generic cascade of linear catalysis reactions. It is observed that all the reactions affect the steady state CV of the last component to similar extent. The steady state FF of the last component is observed to be less sensitive to the first zero order synthesis reaction and is observed to be sensitive to catalysis and degradation reactions to similar extent. The observed distinct sensitivity of CV for transcription or protein degradation reaction is due to characteristic synthesis rate and half-life of mRNA and protein. Local sensitivity analysis revealed that predominantly, the first catalysis reaction maximally affected the steady state CV of the last component while last catalysis reaction maximally affected the FF of the last component.

Overall, by comparing different measures of noise and models of gene expression having different levels of abstractions, the study highlights that the choice of noise measure can affect the conclusions about the effect of reaction parameters on protein variability. It explains the distinct contributions of steps in gene expression to the two measures of noise and provides a comparative view of sensitivity of these measures of noise at steady state protein level over wide parameter ranges. The study also highlights the sensitivity properties of the two measures of noise for generic linear catalysis cascades.

## Supporting Information

S1 File (DOC)
