# Supplementary material for: Ranking of Reactions Based on Sensitivity of Protein Noise Depends on the Choice of Noise Measure
Source: PLoS One. 2015 Dec 1;10(12):e0143867. doi: 10.1371/journal.pone.0143867 (PMC4666593; doi:10.1371/journal.pone.0143867)
Supplement: S1 File — (DOC) [file pone.0143867.s001.doc]

Supplementary Information

Sensitivity scores for various gene expression models

Table A – Average sensitivity score of steady state protein for 4-reaction model of gene expression

| **Step in gene expression** | **Average sensitivity score** | | |
| --- | --- | --- | --- |
|  | **Mean** | **CV** | **Fano Factor** |
| Transcription | 1.53 | 7.07 | 0.32 |
| mRNA degradation | 0.48 | 0.20 | 0.43 |
| Translation | 2.63 | 0.17 | 8.95 |
| Protein degradation | 0.96 | 2.66 | 0.20 |

In the four reaction model the steady state distribution of mRNA is Poisson distribution. For Poisson distribution as the variance and mean are equal the Fano factor is unity. Therefore, the Fano factor should remain unaffected irrespective of parameter values. As expected it was evident from the sensitivity scores that Fano factor is insensitive to transcription and mRNA degradation (Table B). The coefficient of variation at mRNA steady state was observed to be sensitive to transcription much more than to mRNA degradation.

Table B – Average sensitivity score of steady state mRNA for 4-reaction model of gene expression

| **Step in gene expression** | **Average sensitivity score** | | |
| --- | --- | --- | --- |
|  | **Mean** | **CV** | **Fano Factor** |
| Transcription | 8.97 | 6.10 | 0.78 |
| mRNA degradation | 2.03 | 0.45 | 0.78 |

Table C - Average sensitivity score of steady state protein for 6-reaction model of gene expression

| **Step in gene expression** | **Average sensitivity score** | | |
| --- | --- | --- | --- |
|  | **Mean** | **CV** | **Fano Factor** |
| Gene activation | 0.25 | 2.26 | 1.58 |
| Gene deactivation | 0.05 | 0.19 | 0.13 |
| Transcription | 0.33 | 0.43 | 1.58 |
| mRNA degradation | 0.26 | 0.23 | 0.40 |
| Translation | 2.57 | 0.06 | 2.37 |
| Protein degradation | 0.73 | 3.54 | 0.20 |

In case of six reaction model, steady state mRNA distribution differs from Poisson distribution due to addition of gene activation and deactivation reactions. In this case, CV was observed to be most sensitive to gene activation, while Fano factor was observed to be equally sensitive to gene activation and transcription. The absolute values of the sensitivity scores are given in Table D.

TableD - Average sensitivity score of steady state mRNA for 6-reaction model of gene expression

| **Steps in gene expression** | **Average sensitivity score** | | |
| --- | --- | --- | --- |
|  | **Mean** | **CV** | **Fano factor** |
| Gene activation | 0.04 | 1.61 | 3.59 |
| Gene deactivation | 0.08 | 0.11 | 0.22 |
| Transcription | 1.65 | 0.23 | 3.63 |
| mRNA degradation | 1.29 | 0.53 | 0.10 |

In case of 4-reaction model, change in transcription is observed to affect both mean and CV. For 6-reaction model, change in transcription is observed to affect mean and the FF. Change in gene activation is observed to affect both the measures of noise but not the mean.

Supplementary Text

Details of derivation for expression of steady state coefficient of variation and Fano factor in 4-reaction gene expression model

4-reaction gene expression model can be considered as one step catalytic reaction system. Using the framework described in (Gadgil, Lee et al. 2005) the ODEs for time evolution of moments can be obtained as follows.

$$K^{s}=\left[\begin{matrix} ks_{m} & 0 \\ 0 & 0 \end{matrix}\right]$$

$$K^{d}=\left[\begin{matrix} kd_{m} & 0 \\ 0 & kd_{p} \end{matrix}\right]$$

$$K^{cat}=\left[\begin{matrix} 0 & 0 \\ ks_{p} & 0 \end{matrix}\right]$$

$$K=K^{cat}-K^{d}=\left[\begin{matrix} -kd_{m} & 0 \\ ks_{p} & -kd_{p} \end{matrix}\right]$$

The time evolution of mean (M) is given by Eq S1,

$$\frac{dM(t)}{dt}=KM(t)+K^{s}1$$

$$\frac{d}{dt}\left[\begin{matrix} m \\ p \end{matrix}\right]=\left[\begin{matrix} -kd_{m} & 0 \\ ks_{p} & -kd_{p} \end{matrix}\right]\left[\begin{matrix} m \\ p \end{matrix}\right]+\left[\begin{matrix} ks_{m} & 0 \\ 0 & 0 \end{matrix}\right]\left[\begin{matrix} 1 \\ 1 \end{matrix}\right]=\left[\begin{matrix} ks_{m}-kd_{m}.m \\ ks_{p}.m-kd_{p}.p \end{matrix}\right]$$

The time evolution of second moment is given by Eq S3,

$$\frac{dV(t)}{dt}=KV(t)+(Kv(t))^{T}+\Gamma+\Gamma^{T}$$

where $\Gamma_{ij}=\left(K^{cat}_{ij}\_K^{s}_{ii}\right)M_{j}$

$$\frac{dV}{dt}=\left[\begin{matrix} -kd_{m} & 0 \\ ks_{p} & -kd_{p} \end{matrix}\right]\left[\begin{matrix} V_{11} & V_{12} \\ V_{21} & V_{22} \end{matrix}\right]+\left(\left[\begin{matrix} -kd_{m} & 0 \\ ks_{p} & -kd_{p} \end{matrix}\right]\left[\begin{matrix} V_{11} & V_{12} \\ V_{21} & V_{22} \end{matrix}\right]\right)^{T}+\left[\begin{matrix} ks_{m}.m & ks_{m}.p \\ ks_{p}.m & 0 \end{matrix}\right]+\left(\left[\begin{matrix} ks_{m}.m & ks_{m}.p \\ ks_{p}.m & 0 \end{matrix}\right]\right)^{T}$$

$$\frac{dV}{dt}=\frac{d}{dt}\left[\begin{matrix} V_{11} & V_{12} \\ V_{21} & V_{22} \end{matrix}\right]=\left[\begin{matrix} 2\left(ks_{m}.m-kd_{m}.V_{11}\right) & ks_{p}.V_{11}-kd_{p}.V_{21}-kd_{m}.V_{12}+ks_{p}.m+ks_{m}.p \\ ks_{p}.V_{11}-kd_{p}.V_{21}-kd_{m}.V_{12}+ks_{p}.m+ks_{m}.p & 2\left(ks_{p}.V_{12}-kd_{p}.V_{22}\right) \end{matrix}\right]$$

Solving the differential equations for moment at steady state, expressions for the steady state mean and variance for mRNA and protein were obtained.

Mean steady state mRNA level

$$mRNA_{ss}=\frac{ks_{m}}{kd_{m}}$$

Variance at mRNA steady state

$$Var_{mRNA}=\frac{ks_{m}}{kd_{m}}$$

CV of mRNA steady state

$$CV_{mRNA}=\frac{1}{\sqrt{ks_{m}/kd_{m}}}$$

Fano factor of mRNA steady state level

$$FF_{mRNA}=1$$

Analytical expressions for sensitivity

$$\text{Sensitivity of CV}_{\text{mRNA}}\text{ to transcription}=\frac{\delta CV_{mRNA}}{\delta ks_{m}}=-\frac{1}{2}\sqrt{\frac{kd_{m}}{ks_{m}^{3}}}$$

$$\text{Normalised sensitivity = }\frac{\delta CV_{mRNA}}{\delta ks_{m}}.\frac{ks_{m}}{CV_{mRNA}}=-\frac{1}{2}$$

$$\text{Sensitivity of CV}_{\text{mRNA}}\text{ to mRNA degradation}=\frac{\delta CV_{mRNA}}{\delta kd_{m}}=\frac{1}{2\sqrt{ks_{m}kd_{m}}}$$

$$\text{Normalised sensitivity}=\frac{\delta CV_{mRNA}}{\delta kd_{m}}.\frac{kd_{m}}{CV_{mRNA}}=\frac{1}{2}$$

Mean steady state protein level

$$\text{Protein}_{\text{ss}}=\frac{ks_{m}ks_{p}}{kd_{m}kd_{p}}$$

Variance at protein steady state

$$\text{Var}_{\text{Protein}}=\frac{ks_{m}ks_{p}(kd_{m}+ks_{p}+kd_{p})}{kd_{m}kd_{p}(kd_{m}+kd_{p})}$$

CV of protein steady state

$$\text{CV}_{\text{Protein}}=\sqrt{\frac{kd_{m}kd_{p}(kd_{m}+ks_{p}+kd_{p})}{ks_{m}ks_{p}(kd_{m}+kd_{p})}}$$

Fano factor of protein steady state

$$\text{FF}_{\text{Protein}}=\frac{kd_{m}+ks_{p}+kd_{p}}{kd_{m}+kd_{p}}$$

Analytical expressions for sensitivity

$$\begin{matrix} \text{Sensitivity of CV}_{\text{Protein}}\text{ to transcription}=\frac{\delta\text{CV}_{\text{Protein}}}{\delta ks_{m}}=-\frac{kd_{m}kd_{p}(kd_{m}+ks_{p}+kd_{p})}{2ks_{m}^{2}ks_{p}(kd_{m}+kd_{p})\sqrt{\frac{kd_{m}kd_{p}(kd_{m}+ks_{p}+kd_{p})}{ks_{m}ks_{p}(kd_{m}+kd_{p})}}} \\ \text{ }=-\frac{1}{2}\sqrt{\frac{kd_{m}kd_{p}(kd_{m}+ks_{p}+kd_{p})}{ks_{m}^{3}ks_{p}(kd_{m}+kd_{p})}} \end{matrix}$$

$$\text{Normalised sensitivity}=\frac{\delta\text{CV}_{\text{Protein}}}{\delta ks_{m}}.\frac{ks_{m}}{\text{CV}_{\text{Protein}}}=-\frac{1}{2}$$

$$\begin{matrix} \text{Sensitivity of CV}_{\text{Protein}}\text{ to mRNA degradation=}\frac{\delta CV_{\text{Protein}}}{\delta kd_{m}}=\frac{\frac{kd_{m}kd_{p}}{ks_{m}ks_{p}(kd_{m}+kd_{p})}-\frac{kd_{m}kd_{p}(kd_{m}+ks_{p}+kd_{p})}{ks_{m}ks_{p}(kd_{m}+kd_{p})^{2}}+\frac{kd_{p}(kd_{m}+ks_{p}+kd_{p})}{ks_{m}ks_{p}(kd_{m}+kd_{p})}}{2\sqrt{\frac{kd_{m}kd_{p}(kd_{m}+ks_{p}+kd_{p})}{ks_{m}ks_{p}(kd_{m}+kd_{p})}}} \\ \text{ }=\frac{kd_{p}\left(kd_{m}^{2}+2kd_{m}kd_{p}+kd_{p}^{2}+ks_{p}kd_{p}\right)}{2\sqrt{ks_{m}kd_{m}ks_{p}kd_{p}(kd_{m}+kd_{p})^{3}(kd_{m}+ks_{p}+kd_{p})}} \end{matrix}$$

$$\text{Normalised sensitivity}=\frac{\delta CV_{\text{Protein}}}{\delta kd_{m}}.\frac{kd_{m}}{CV_{\text{Protein}}}=\frac{kd_{m}^{2}+2kd_{m}kd_{p}+kd_{p}^{2}+ks_{p}kd_{p}}{2(kd_{m}+kd_{p})(kd_{m}+ks_{p}+kd_{p})}$$

$$\begin{matrix} \text{Sensitivity of CV}_{\text{Protein}}\text{ to translation=}\frac{\delta CV_{\text{Protein}}}{\delta ks_{p}}=\frac{\frac{kd_{m}kd_{p}}{ks_{m}kd_{p}(kd_{m}+kd_{p})}-\frac{kd_{m}kd_{p}(kd_{m}+ks_{p}+kd_{p})}{ks_{m}ks_{p}^{2}(kd_{m}+kd_{p})}}{2\sqrt{\frac{kd_{m}kd_{p}(kd_{m}+ks_{p}+kd_{p})}{ks_{m}ks_{p}(kd_{m}+kd_{p})}}} \\ \text{ }=-\frac{1}{2}\sqrt{\frac{kd_{m}kd_{p}(kd_{m}+kd_{p})}{ks_{m}ks_{p}(kd_{m}+ks_{p}+kd_{p})}} \end{matrix}$$

$$\text{Normalised sensitivity}=\frac{\delta CV_{\text{Protein}}}{\delta ks_{p}}.\frac{ks_{p}}{CV_{\text{Protein}}}=\frac{-(kd_{m}+kd_{p})}{2(kd_{m}+ks_{p}+kd_{p})}$$

$$\text{Normalised sensitivity}=\frac{\delta CV_{\text{Protein}}}{\delta kd_{p}}.\frac{kd_{p}}{\text{CV}_{\text{Protein}}}=\frac{kd_{m}^{2}+2kd_{m}kd_{p}+kd_{p}^{2}+ks_{p}kd_{m}}{2(kd_{m}+kd_{p})(kd_{m}+ks_{p}+kd_{p})}$$

$$\text{Normalised sensitivity}=\frac{\delta FF_{\text{Protein}}}{\delta kd_{m}}.\frac{kd_{m}}{FF_{\text{Protein}}}=\frac{-kd_{m}ks_{p}}{(kd_{m}+kd_{p})(kd_{m}+ks_{p}+kd_{p})}$$

$$\text{Sensitivity of FF}_{\text{Protein}}\text{ to translation = }\frac{\delta\text{FF}_{\text{Protein}}}{\delta ks_{p}}=\frac{1}{kd_{m}+kd_{p}}$$

$$\text{Normalised sensitivity}=\frac{\delta FF_{\text{Protein}}}{\delta ks_{p}}.\frac{ks_{p}}{FF_{\text{Protein}}}=\frac{ks_{p}}{kd_{m}+ks_{p}+kd_{p}}$$

$$\text{Normalised sensitivity}=\frac{\delta\text{FF}_{\text{Protein}}}{\delta kd_{p}}.\frac{kd_{p}}{\text{FF}_{\text{Protein}}}=\frac{-kd_{p}ks_{p}}{(kd_{m}+kd_{p})(kd_{m}+ks_{p}+kd_{p})}$$

Details of derivation of ordinary differential equations for time evolution of moments for 6-reaction model of gene expression

$$K_{6}^{s}=0$$

$$K_{6}^{d}=\left[\begin{matrix} 0 & 0 & 0 & 0 \\ 0 & 0 & 0 & 0 \\ 0 & 0 & kd_{m} & 0 \\ 0 & 0 & 0 & kd_{p} \end{matrix}\right]$$

$$K_{6}^{cat}=\left[\begin{matrix} 0 & 0 & 0 & 0 \\ 0 & 0 & 0 & 0 \\ 0 & ks_{m} & 0 & 0 \\ 0 & 0 & ks_{p} & 0 \end{matrix}\right]$$

$$K_{6}^{con}=\left[\begin{matrix} -k_{on} & k_{off} & 0 & 0 \\ k_{on} & -k_{off} & 0 & 0 \\ 0 & 0 & 0 & 0 \\ 0 & 0 & 0 & 0 \end{matrix}\right]$$

$$K_{6}=K_{6}^{cat}+K_{6}^{con}-K_{6}^{d}=\left[\begin{matrix} -k_{on} & k_{off} & 0 & 0 \\ k_{on} & -k_{off} & 0 & 0 \\ 0 & ks_{m} & -kd_{m} & 0 \\ 0 & 0 & ks_{p} & -kd_{p} \end{matrix}\right]$$

The time evolution of mean (M_6_) was given as,

$$\frac{dM_{6}(t)}{dt}=\frac{d}{dt}\left[\begin{matrix} g_{off} \\ g_{on} \\ m \\ p \end{matrix}\right]=K_{6}M_{6}(t)=\left[\begin{matrix} -k_{on} & k_{off} & 0 & 0 \\ k_{on} & -k_{off} & 0 & 0 \\ 0 & ks_{m} & -kd_{m} & 0 \\ 0 & 0 & ks_{p} & -kd_{p} \end{matrix}\right]\left[\begin{matrix} g_{off} \\ g_{on} \\ m \\ p \end{matrix}\right]=\left[\begin{matrix} -k_{on}.g_{off}+k_{off}.g_{on} \\ k_{on}.g_{off}-k_{off}.g_{on} \\ ks_{m}.g_{on}-kd_{m}.m \\ ks_{p}.m-kd_{p}.p \end{matrix}\right]$$

The time evolution of second moment was given as,

$$\frac{dV_{6}(t)}{dt}=K_{6}V_{6}(t)+(K_{6}V_{6}(t))^{T}+\Gamma_{6}+\Gamma_{6}^{T}$$

where

$$\begin{matrix} \frac{dV_{6}(t)}{dt}=\frac{d}{dt}\left[\begin{matrix} V_{11} & V_{12} & V_{13} & V_{14} \\ V_{21} & V_{22} & V_{23} & V_{24} \\ V_{31} & V_{32} & V_{33} & V_{34} \\ V_{41} & V_{42} & V_{43} & V_{44} \end{matrix}\right]=\left[\begin{matrix} -k_{on} & k_{off} & 0 & 0 \\ k_{on} & -k_{off} & 0 & 0 \\ 0 & ks_{m} & -kd_{m} & 0 \\ 0 & 0 & ks_{p} & -kd_{p} \end{matrix}\right]\left[\begin{matrix} V_{11} & V_{12} & V_{13} & V_{14} \\ V_{21} & V_{22} & V_{23} & V_{24} \\ V_{31} & V_{32} & V_{33} & V_{34} \\ V_{41} & V_{42} & V_{43} & V_{44} \end{matrix}\right]+(K_{6}V_{6}(t))^{T} \\ +\left[\begin{matrix} 0 & 0 & 0 & 0 \\ 0 & 0 & 0 & 0 \\ 0 & ks_{m}.g_{on} & 0 & 0 \\ 0 & 0 & ks_{p}.m & 0 \end{matrix}\right]+\left[\begin{matrix} 0 & 0 & 0 & 0 \\ 0 & 0 & ks_{m}.g_{on} & 0 \\ 0 & 0 & 0 & ks_{p}.m \\ 0 & 0 & 0 & 0 \end{matrix}\right] \end{matrix}$$

Global sensitivity analysis of generic linear catalysis cascade

Tables showing global sensitivity scores

| **2 Component** | **Parameter** | **Average Score** |
| --- | --- | --- |
| **%CV** | **ks** | 1.27 |
|  | **kcat1** | 1.61 |
|  | **kd1** | 1.04 |
|  | **kd2** | 1.62 |
| **FF** | **ks** | 0.14 |
|  | **kcat1** | 2.53 |
|  | **kd1** | 0.70 |
|  | **kd2** | 1.23 |

| **3 Component** | **Parameter** | **Average Score** |
| --- | --- | --- |
| **%CV** | **ks** | **0.81** |
|  | **kcat1** | **1.06** |
|  | **kcat2** | **0.33** |
|  | **kd1** | **0.88** |
|  | **kd2** | **0.73** |
|  | **kd3** | **0.87** |
| **FF** | **ks** | **0.28** |
|  | **kcat1** | **0.70** |
|  | **kcat2** | **1.05** |
|  | **kd1** | **0.89** |
|  | **kd2** | **0.86** |
|  | **kd3** | **0.52** |

| **4 Component** | **Parameter** | **Average Score** |
| --- | --- | --- |
| **%CV** | **ks** | **0.65** |
|  | **kcat1** | **0.60** |
|  | **kcat2** | **0.93** |
|  | **kcat3** | **0.52** |
|  | **kd1** | **0.49** |
|  | **kd2** | **0.98** |
|  | **kd3** | **0.28** |
|  | **kd4** | **0.47** |
| **FF** | **ks** | **0.30** |
|  | **kcat1** | **0.48** |
|  | **kcat2** | **0.79** |
|  | **kcat3** | **0.57** |
|  | **kd1** | **0.31** |
|  | **kd2** | **0.30** |
|  | **kd3** | **0.64** |
|  | **kd4** | **0.31** |

| **5 Component** | **Parameter** | **Average Score** |
| --- | --- | --- |
| **%CV** | **ks** | **0.23** |
|  | **kcat1** | **0.27** |
|  | **kcat2** | **0.52** |
|  | **kcat3** | **0.69** |
|  | **kcat4** | **0.35** |
|  | **kd1** | **0.46** |
|  | **kd2** | **0.40** |
|  | **kd3** | **0.81** |
|  | **kd4** | **0.74** |
|  | **kd5** | **0.44** |
| **FF** | **ks** | **0.33** |
|  | **kcat1** | **0.44** |
|  | **kcat2** | **0.41** |
|  | **kcat3** | **0.39** |
|  | **kcat4** | **0.80** |
|  | **kd1** | **0.40** |
|  | **kd2** | **0.45** |
|  | **kd3** | **0.59** |
|  | **kd4** | **0.85** |
|  | **kd5** | **0.90** |

| **6 Component** | **Parameter** | **Average Score** |
| --- | --- | --- |
| **%CV** | **ks** | **0.68** |
|  | **kcat1** | **0.33** |
|  | **kcat2** | **0.28** |
|  | **kcat3** | **0.65** |
|  | **kcat4** | **0.75** |
|  | **kcat5** | **0.44** |
|  | **kd1** | **0.31** |
|  | **kd2** | **0.85** |
|  | **kd3** | **0.47** |
|  | **kd4** | **0.24** |
|  | **kd5** | **0.75** |
|  | **kd6** | **0.87** |
| **FF** | **ks** | **0.46** |
|  | **kcat1** | **0.21** |
|  | **kcat2** | **0.55** |
|  | **kcat3** | **0.69** |
|  | **kcat4** | **0.67** |
|  | **kcat5** | **0.33** |
|  | **kd1** | **0.26** |
|  | **kd2** | **0.71** |
|  | **kd3** | **0.95** |
|  | **kd4** | **0.24** |
|  | **kd5** | **0.44** |
|  | **kd6** | **0.41** |

Local sensitivity analysis of generic linear catalysis cascade

Local sensitivity analysis was performed as mentioned in the Methodology section in main text. At each data point the reactions are ranked according to the local sensitivity coefficient. The frequency of each reaction having highest rank was calculated. The obtained frequency was compared with the expected equal frequency, in order to test the null hypothesis that all the reactions affect the output (coefficient of variation or Fano factor) to equal extent. The statistics of chi-square test of independence is given in Table. Use of different number of samples is to ensure the expected frequency of each reaction type equal to 10, i.e. greater than the minimum requirement of 5.

| **2 component** | **Observed frequency** | |
| --- | --- | --- |
| **Parameter** | **CV** | **FF** |
| **kcat1** | 3 | 28 |
| **kd1** | 12 | 2 |
| **kd2** | 15 | 0 |
| **Number of samples** | 30 | 30 |
| **chisq** | 7.8 | 48.8 |
| **df** | 2 | 2 |
| **p-val** | 0.02024 | 2.53E-11 |

| **3 component** | **Observed frequency** | |
| --- | --- | --- |
| **Parameter** | **CV** | **FF** |
| **kcat1** | 26 | 0 |
| **kcat2** | 0 | 31 |
| **kd1** | 13 | 0 |
| **kd2** | 4 | 11 |
| **kd3** | 7 | 8 |
| **Number of samples** | 50 | 50 |
| **chisq** | 41 | 64.6 |
| **df** | 4 | 4 |
| **p-val** | 2.69E-08 | 3.12E-13 |

| **4 component** | **Observed frequency** | |
| --- | --- | --- |
| **Parameter** | **CV** | **FF** |
| **kcat1** | 34 | 0 |
| **kcat2** | 0 | 0 |
| **kcat3** | 0 | 41 |
| **kd1** | 14 | 0 |
| **kd2** | 6 | 1 |
| **kd3** | 9 | 14 |
| **kd4** | 7 | 14 |
| **Number of samples** | 70 | 70 |
| **chisq** | 81.8 | 137.4 |
| **df** | 6 | 6 |
| **p-val** | 1.52E-15 | < 2.2e-16 |

| **5 component** | **Observed frequency** | |
| --- | --- | --- |
| **Parameter** | **CV** | **FF** |
| **kcat1** | 54 | 0 |
| **kcat2** | 0 | 0 |
| **kcat3** | 0 | 0 |
| **kcat4** | 0 | 58 |
| **kd1** | 25 | 0 |
| **kd2** | 1 | 1 |
| **kd3** | 2 | 3 |
| **kd4** | 4 | 17 |
| **kd5** | 4 | 11 |
| **Number of samples** | 90 | 90 |
| **chisq** | 267.8 | 288.4 |
| **df** | 8 | 8 |
| **p-val** | < 2.2e-16 | < 2.2e-16 |

| **6 component** | |  |
| --- | --- | --- |
| **Parameter** | **CV** | **FF** |
| **kcat1** | 62 | 0 |
| **kcat2** | 0 | 0 |
| **kcat3** | 0 | 0 |
| **kcat4** | 0 | 0 |
| **kcat5** | 0 | 50 |
| **kd1** | 15 | 0 |
| **kd2** | 8 | 0 |
| **kd3** | 7 | 1 |
| **kd4** | 6 | 1 |
| **kd5** | 7 | 24 |
| **kd6** | 5 | 34 |
| **Number of samples** | 110 | 110 |
| **chisq** | 319.2 | 313.4 |
| **df** | 10 | 10 |
| **p-val** | < 2.2e-16 | < 2.2e-16 |
